# Exercise Training-Induced Extracellular Matrix Protein Adaptation in Locomotor Muscles: A Systematic Review

**DOI:** 10.3390/cells10051022

**Published:** 2021-04-26

**Authors:** Efpraxia Kritikaki, Rhiannon Asterling, Lesley Ward, Kay Padget, Esther Barreiro, Davina C. M. Simoes

**Affiliations:** 1 Faculty of Health and Life Sciences, Northumbria University Newcastle, Newcastle upon Tyne NE1 8ST, UK; effie.kritikaki@northumbria.ac.uk (E.K.); rhiannon.ast@gmail.com (R.A.); lesley.ward@northumbria.ac.uk (L.W.); kay.padget@northumbria.ac.uk (K.P.); 2 Pulmonology Department, Lung Cancer and Muscle Research Group, Hospital del Mar-IMIM, Parc de Salut Mar, Health and Experimental Sciences Department (CEXS), Universitat Pompeu Fabra (UPF), CIBERES, 08002 Barcelona, Spain; ebarreiro@imim.es

**Keywords:** extracellular matrix, skeletal muscle, glycoproteins, proteoglycans, collagens, exercise training, ageing, remodelling, adaptation, myogenesis

## Abstract

Exercise training promotes muscle adaptation and remodelling by balancing the processes of anabolism and catabolism; however, the mechanisms by which exercise delays accelerated muscle wasting are not fully understood. Intramuscular extracellular matrix (ECM) proteins are essential to tissue structure and function, as they create a responsive environment for the survival and repair of the muscle fibres. However, their role in muscle adaptation is underappreciated and underinvestigated. The PubMed, COCHRANE, Scopus and CIHNAL databases were systematically searched from inception until February 2021. The inclusion criteria were on ECM adaptation after exercise training in healthy adult population. Evidence from 21 studies on 402 participants demonstrates that exercise training induces muscle remodelling, and this is accompanied by ECM adaptation. All types of exercise interventions promoted a widespread increase in collagens, glycoproteins and proteoglycans ECM transcriptomes in younger and older participants. The ECM controlling mechanisms highlighted here were concerned with myogenic and angiogenic processes during muscle adaptation and remodelling. Further research identifying the mechanisms underlying the link between ECMs and muscle adaptation will support the discovery of novel therapeutic targets and the development of personalised exercise training medicine.

## 1. Introduction

Skeletal muscle mass accounts for 40% of total body mass. Muscle is a highly plastic tissue, and adaptation is seen after increased locomotory and metabolic demands of exercise training. Adaptation in terms of altered muscle physiology and improved performance varies greatly according to the activities imposed, such as force, duration, as well as individual’s capacity to respond, which is controlled by their genetic makeup [1].

Ageing is associated with progressive decline in skeletal muscle mass, muscle strength and regenerative capacity [2]. Weakened muscles increase the likelihood of injury and ineffective repair processes, negatively affecting quality of life. Exercise training is applied as a therapeutic intervention capable of improving aged muscle regeneration, muscle strength and muscle mass [3,4,5]. Most of the investigations have traditionally focused on elucidating the phenotypic changes on muscle strength, size and fibre type distribution. At the cellular and molecular level, several markers of cellular anabolism and catabolism have been investigated [2,3,6]. However, studies on the effect of exercise training promoting muscle extracellular matrix (ECM) adaptation are limited and have been frequently overlooked.

The intramuscular ECM is widely distributed throughout muscle tissue, maintaining its structure. The ECM endomysium embeds the individual muscle fibres and the neighbouring myofibers are organized into fascicles encased by the perimysium. The whole muscle is ensheathed by another layer of ECM connective tissue named the epimysium [7]. Intramuscular ECMs provide mechanical support to muscle tissue, nerves and blood vessels. Recent research has demonstrated that the ECM plays an important role in muscle growth [8] and repair processes [9], as well as the transmission of contractile force [10]. Nevertheless, the role of ECM adaptation on muscle regeneration and remodelling after exercise training stimulus is still unclear. The intramuscular ECM adaptation seen during ageing has been reviewed elsewhere [11].

As a post-mitotic tissue, the homeostasis of the skeletal muscles depends on the capacity of the satellite cells to adapt and regenerate. Under normal conditions, adult muscle turnover as result of daily life activities relies on sporadic proliferation and fusion of satellite cells to muscle fibres. Satellite cells are found in a cell niche, which consists of a mesh of components containing a mixture of glycoproteins and proteoglycans and growth factors. The ECMs in the niche provide a dynamic environment, transmitting mechanical and biochemical signals. The function of these ECM molecules is to maintain satellite cell quiescence, activation, proliferation and differentiation [9,11,12].

Exercise training is the most potent strategy to improve muscle fibre cross-sectional area. This intervention remodels peripheral skeletal muscle architecture and alters the ECM expression involved in this process [13,14,15]. However, the extent to which ECMs contribute to the regeneration and remodelling of the skeletal muscle upon exercise training is still unclear. The aim of this systematic review is to provide up-to-date information on the effect of exercise training inducing ECM adaptation and the involvement of ECMs on skeletal muscle remodelling.

## 2. Materials and Methods

This systematic review was compiled using guidelines described in the Preferred Reporting Items for Systematic Reviews and Meta-Analyses (PRISMA) [16]. The review protocol is registered with the International Prospective Register of Systematic Reviews (PROSPERO ID: CRD42021206259).

### 2.1. Search Strategy

A literature search of online databases, PubMed, Cochrane Central Register of Controlled Trials (CENTRAL), Scopus, and CINAHL was conducted from their inception to March 2021. These databases were chosen due to their relevance in the field, use in other systematic reviews [17,18,19] and in consultation with a university librarian. All databases were accessed via the Northumbria University library platform. A search on PEDro database did not retrieve study results framework. In addition to the database search, citations from studies in the field were screened for the criteria and inserted as other sources.

To develop the search strategy, relevant terminology was determined from published reviews associated with ECM in skeletal muscle and with exercise training [11,20]. Following this, search strings based on a combination of a mix of Medical Subject Headings (MeSH) or free text words related to “extracellular matrix proteins”, “exercise”, “activity”, “skeletal muscle remodelling”, “cachexia” and “skeletal muscle wasting” were used to form the PICO (population, intervention, comparators, outcome) framework (Supplementary Table S1).

### 2.2. Study Selection and Data Extraction

Peer reviewed studies published in the English language and involving healthy human participants were eligible to be included in this systematic review. Studies evaluating the effect of exercise training intervention on primary or secondary outcome demonstrating ECM expression at mRNA and protein level were included. ECM-related molecules were not part of the outcome search. No chronological and study design limitations were determined.

Studies exclusively testing animal or in vitro models were excluded from this systematic review. Studies describing immobilization and/or disuse interventions, as well as studies testing the effect of a single bout of exercise were excluded. Other studies excluded were those that analysed non-peripheral skeletal muscle, pharmacological interventions, and those on neuromuscular pathologies. Studies that did not contain any ECM marker in their outcomes were also excluded from the current review.

Selected outcomes were exported to EndNote software (Thomson Reuters, New York, NY, USA). Duplicates were removed using the systematic review management software program Rayyan (Qatar Computing Research Institute, Doha, Qatar). Titles and abstracts were screened by two reviewers (E.K. and R.A.), articles were fully text read and assessed for inclusion eligibility. Any disputes were resolved by a third reviewer (D.C.M.S.). Data were extracted individually by two reviewers (E.K. and R.A.).

### 2.3. Quality Assessment

The outcomes were assessed for risk of bias (E.K., R.A.), and discrepancies resolved (E.K.). As the studies included randomized controlled trials (RCTs) or non-RCTs, the revised versions of Cochrane Collaboration’s tool (RoB2) and ROBINS-I were used to assess the risk of bias, respectively [21,22,23]. ROBINS-I is the preferentially recommended tool to evaluate the risk of bias of non-RCTs [23]. This tool was specifically developed to evaluate the risk of bias estimating the comparative effectiveness of interventions in studies not adopting randomisation in allocating units (individual or cluster of individuals) into comparison groups [21]. Additionally, bias domains that are included in ROBINS-I considered the pre-intervention, at-intervention and post-intervention periods. Whereas RoB2 is a suitable tool for assessing the risk of bias for RCTs [23]. RoB2 domains of assessing bias included signalling questions that were based on the randomisation process, deviations from the intended interventions, missing outcome data, missing reported results, selections of the reported results and an overall bias [22]. The interpretation of the domain-level and overall risk of bias judgements in both assessment tools are “Low risk”, “Moderate risk”, “Serious risk” and “Critical risk” of bias, which were calculated from the checklist scores according to ROBINS-I and RoB2 guidance [21,22]

## 3. Results

**Figure 1:**
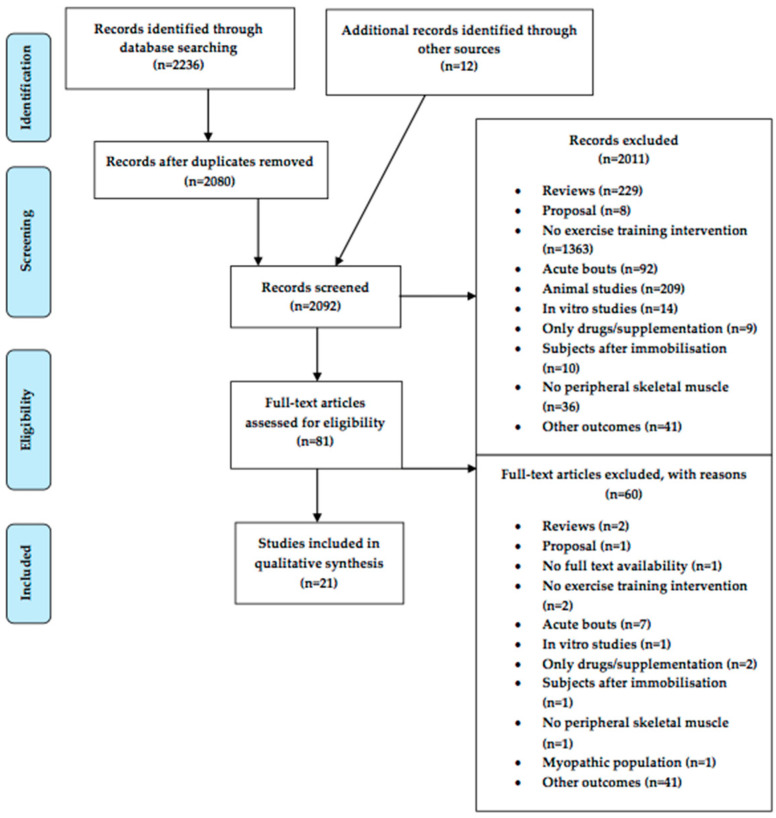
PRISMA flow diagram of the identification and selection process.

The systematic search of the online database identified 2236 articles. Twelve articles were added from other sources. All articles were screened using the eligibility and exclusion criteria. The PRISMA flow diagram (Figure 1) summarizes the search strategy and the study selection process.

### 3.1. Analysis of Risk of Bias

Of the 21 studies included, the overall bias of 13 studies was moderate, in 7 of them it was low and 1 of them it was high. The bias sections where the outcome was not clearly stated, they were marked as “unclear”. The risk of bias classification for each article using the ROBINS-I and ROB2 tools are displayed in Table 1 and Table 2, respectively.

### 3.2. Study and Subject Characteristics

Of the 21 studies included in the systematic review, 18 were non-RCT of pre–post study design [24,25,26,27,28,29,30,31,32,33,34,35,36,37,38,39,40,41] and 3 RCTs [42,43,44]. Even though our systematic review focuses on healthy subjects, two studies were on clinical populations [34,44]. However, no fibrosis or other neuromuscular pathologies were observed in these studies. The exercise training interventions prescribed were aerobic exercise training (AET) [30,31,35,37,39,40,41,44], resistance training (RET) [24,25,28,33,34,36,42,43], combined aerobic and resistance training (CT) [26,27,32], electrical stimulation (ES) [29] and one comparing resistance training, high-intensity interval training (HIIT) and combined training after a sedentary period [38]. The duration of the exercise interventions ranged from 5 to 13 weeks, the frequency was 2 to 9 sessions per week, and the mean calculated (duration x number of sessions) dosage was 35.3 hrs. We were able to accurately estimate dosage in 18 out of the 21 studies included. Notably, the training duration was different among age groups. In older age populations, longer training intervention for more than 9 weeks was preferably prescribed, whereas in the younger age groups, the training tended to be shorter than 9 weeks. The characteristics of the studies’ interventions are presented in Table 3, with the clinical studies placed at bottom of the Tables. No adverse events were mentioned throughout the duration of these studies.

A total of 402 healthy individuals, with an average age of 46.8 years old (19–77.4 years old) and 85 individuals from a clinical population with an average age 57.9 years participated across the studies. The majority of studies included only male participants representing 69% of the total population, four studies included males and females [29,36,38,41], and two studies exclusively females [34,43]. Among the healthy individuals, 69% were male and 31% female. The majority of the studies involved either a young group (<38 years old) with an average age 25.1 ± 1.8 years old or an old group with an average age 65.7 ± 9.9 years old. Direct comparison of the effect of exercise on ECMs in young and old populations was limited to data from only two studies [36,38]. A summary of participants’ characteristics and physiological changes as result of exercise intervention is shown in Table 3.

### 3.3. Exercise Training Increases the Expression of ECMs Associated with Skeletal Muscle Remodelling

Muscle adaptations were quantified in several studies and are presented in Table 3 as muscle strength, body composition and muscle architecture. The changes in muscle strength and capacity were demonstrated as significant increase in leg and knee extension strength, peak torque and grip strength [26,28,29,34,36,38,42,43]. Body composition with increased body fat free mass (FFM), lean body mass (LBM), and decreased percentage of fat was also observed in participants after various exercise training interventions [26,28,31,37,38,42,43]. Muscle architecture also changed significantly with exercise intervention increasing muscle cross-sectional area, fibre size, capillary-to-fibre ratio [26,28,36,40,42]. The studies analysing muscle remodelling and adaptation demonstrated that the exercise training interventions were adequate and sufficient to promote peripheral skeletal muscle changes in both age groups (Table 3). The phenotypical changes in the muscle were accompanied by widespread increase in the expression of ECMs at mRNA and protein level, as listed in Table 4. Among the 54 ECMs reported at the mRNA level, only glypican 4, chondroadherin and LAMβ3 were recoded to be downregulated in older participants. In addition, decorin was downregulated only in young participants when training duration was 12 weeks, otherwise upregulated in all the other conditions tested. At the protein level, 21 ECMs were significantly upregulated in total muscle tissue after exercise training [25,30,40]; however, dermatopontin, irisin, laminin subunit alpha 4 were significantly reduced. Seven studies provide data on direct association of ECM expression and muscle remodelling, which is represented in Table 4. Our findings demonstrate that exercise training induces muscle remodelling as well as ECM changes at transcription and translation levels.

**Figure 2:**
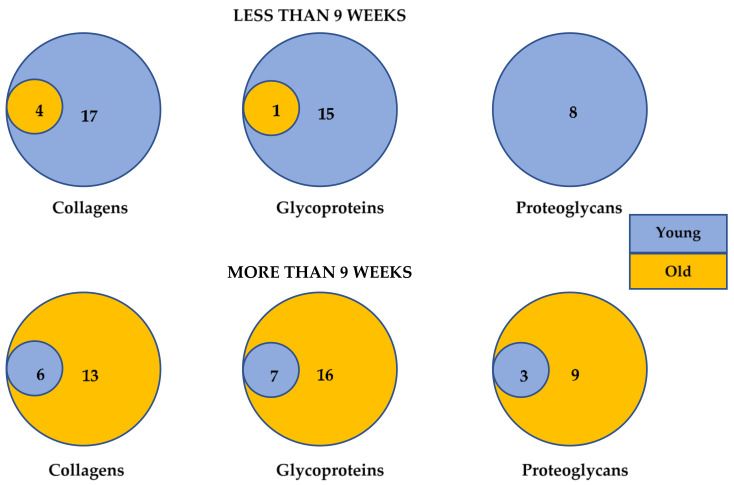
Venn diagrams of the upregulated collagens, proteoglycans and glycoproteins ECMs transcriptomes in healthy young and old participants when exercise training lasted less than or more than 9 weeks.

The duration of exercise training was different among age groups. The exercise duration prescribed to younger participants tended to be less than 9 weeks, whereas for older participants training lasted more than 9 weeks. Figure 2 illustrates the overlap in ECMs transcriptomes categorised as collagens, glycoproteins and proteogycans upregulated in young and old populations, when duration of intervention was less and more than 9 weeks. Similar transcriptomes per ECM category were reported in both age groups independent of training duration. The upregulation of collagens, glycoproteins and proteoglycans ECMs were observed across all types of exercise training interventions. Despite the tendency of prescribing AET and HIIT to young participants, and CT to older age groups, similar ECM transcriptomes were reported in both age groups. When comparing the effect of exercise training modalities, Robinson et al. [38] demonstrated that all types of training including CT, RET and HIIT induced similarly ECM transcriptomes in both age groups. However, COL14α1, lumican and elastin were the exceptions. The HIIT training upregulated these transcriptomes only in the older participants.

ECM-related molecules were also reported in some of the studies. As they were not part of the outcome search, they were not analysed in this systematic review, but a list is provided in Supplementary Table S2.

### 3.4. ECM Adaptation Associated with Muscle Structure and Stability

ECMs play a crucial role in muscle structure. They stabilise muscle cells by modifying the mechanical properties of the tissue, decreasing its stress and making it more load resistant. The failure of ECMs to maintain this structure results in increased susceptibility to mechanical stress and muscular fibre necrosis. Collagen fibrils provide mechanical stability to the skeletal muscle and regulate cell adhesion and differentiation. They confer tensile strength, rigidity, and compliance to the muscle [11,45]. Among the collagen superfamily, collagen I and III are present in the form of fibrils and they account for the 75% of the total skeletal muscle collagen. Exercise training promoted a widespread increase in mRNA expression of various types of collagen including COL1, COL3, COL4, COL5, COL6, COL8, COL12, COL14, COL15, and COL18 and PLOD2 in both age groups [29,30,37,39]. The increase in collagens is also shown at protein level [30], demonstrating an increase in posttranscriptional and translation events. The studies comparing younger to older participants [36,38] demonstrated a higher fold change in ECMs from older participants, which was dependent on the type of exercise modality. Robinson et al. found that older participants present greater induction of the transcriptomes for collagen IV, collagen XIV, lumican, elastin and periostin after HIIT training, but not after RT and CT [38], whereas Raue et al. demonstrate that RT induces a greater fold change of collagens, proteoglycans and glycoproteins in older participants compared to young [36].

The collagen types XV and XVIII have structural features of both collagens and proteoglycans [46]. These collagens are known to be associated with the stability of micro-vessels to muscle cells [47] and they were transcriptionally upregulated mainly in older subjects after CT or RET [26,36] and in young after AET [30,39].

Type IV collagen is the major constituent of the basement membrane supporting the ECM niche for satellite cells. Collagen IV forms a complex network tethering other ECM proteins, including laminins and proteoglycans, as well as growth factors and cellular receptors, as reviewed elsewhere [48]. COL4 mRNA expression was increased after all types of exercise training and in subjects of all ages at mRNA and protein level [24,26,30,36,37,38], including after shorter durations of AET [37]. Interestingly, HIIT induced a more than 60% increase in COL4 in older participants compared to young [38].

Among the glycoproteins, the laminin subunits were significantly increased across all the population tested at the level of mRNA [25,26,30,36,38,39] and protein [24]. Laminins are heterodimers constituted by association of three different gene products, the α, β and γ chains. LAMA4, LAMB1, LAMC1 and LAMC3 transcriptomes were upregulated after exercise, but LAMβ3 chain was decreased in older subjects after CT [26]. At the basement membrane, laminin serves as a ligand for the sarcolema receptors of the dystrophyn-associated glycoprotein complex and the α7β1 integrin. In addition to its central role in the architecture and stability of the basement membrane, laminins control and trigger cellular functions by interacting with cell surface components and trapping growth factors [49]. Nidogens also contribute to the structural support of the muscle by promoting the interactions between laminin and collagens [48]. All types of exercise training significantly induced upregulation in the mRNA expression of nidogen 1 and 2, irrespective of participants’ age [26,36,38,39].

Perlecan (HSPG2) is a pericellular proteoglycan found at the basement membrane and it was reportedly increased in two studies investigating young participants after AET [30,39] and in one study in older adults after CT [26]. Proteoglycans interact with elastic fibres providing tissue extensibility and resilience [50]. At transcriptional level, CT and HIIT increased elastin and elasticity-associated emilin-3 in older participants [26,38].

### 3.5. ECMs Associated with Myogenic Regeneration and Repair

A similar number of glycoproteins were identified to be upregulated in both age groups after exercise training. However, fibronectin, tenascin C and fibrillin were only reported in the young group [33,39,40]. The adhesive properties of these glycoproteins are crucial to satellite cell function promoting muscle repair and regeneration. It is unknown why the retrieved studies did not report these glycoproteins in the muscle of older participants. One of the studies measuring serum fibronectin in older diabetic patients (48.8 ± 14.6 years old) found a 50% reduction of this protein in response to moderate aerobic training [44].

Osteonectin (SPARC) was one of the most frequently reported glycoproteins in the retrieved studies. SPARC was increased after all types of exercise stimuli, duration and age groups [26,32,34,36,37,38,39,41]. This matricellular protein is essential to tissue regeneration by contributing to myofiber metabolic homeostasis, reduction in inflammation, extracellular matrix remodelling and collagen maturation [51].

Proteoglycans are also involved in the process of skeletal muscle regeneration [52] and are upregulated after skeletal muscle damage in newly formed myotubes [53]. The mRNA expression of the small leucine-rich repeat proteoglycans (SLRPs), biglycan, asporin, osteoglycin (or mimecan), lumican, was upregulated in all age groups after various exercise interventions and durations [26,30,33,36,38,39]. At protein level, AET increased the expression of asporin, lumican and prolargin in young participants [30]. Decorin is the most abundant proteoglycan in the skeletal muscle and was reported in both age groups after training. However, in young participants, decorin was either unchanged or downregulated upon the stimulus of AET [30,31], whereas in older participants it was upregulated following a CT intervention [27] and RET in osteoporotic women [34]. The proteoglycans perlecan (HSPG2) and ECM2 were reported to be upregulated independent of age or intervention type [26,30,38,39]. Aggrecan (CSPG4) was reported to be increased when measured after CT in older participants [26]. Versican (CSPG2) was reportedly increased only after AET in young participants [39].

Glypican 4 and chondroadherin are proteoglycans reported only in the older group and were among the few ECMs downregulated after exercise training [26], while in the same study, osteomodulin and phosphacan/receptor-type protein phosphatase *β* (CSPG4) [26] were induced by CT exercise in old participants. The cell surface proteoglycan CSPG4 interacts with neurons and neural cell-adhesion molecules (N-CAM) in addition to blocking N-CAM and tenascin growth-promoting ability [46].

### 3.6. Angiogenesis

Increased capillarisation to muscle fibre ratio was seen after exercise training across age groups and a range of body mass indexes (BMI) [4,6,54]. Adhesive glycoproteins such as thrombospondins are involved in angiogenesis [55]. Combined exercise training enhanced the mRNA expression of both the anti-angiogenic thrombospondin 1 (THBS1) and the pro-angiogenic thrombospondin 4 (THBS4), but the THBS4/THBS1 fold change ratio was 1.4 [26]. Although tenascin C is upregulated as consequence of injured muscle fibre [56] and damaging eccentric contraction [57], Valdivieso et al. [40] testing young participants after AET demonstrated that A/A genotype has superior gain in muscle capillarization postexercise, as compared to the T/T genotype Tenascin C (Tn-C). In addition, exercise training upregulated perlecan in both age groups [26,30,39], indicating a pro-angiogenic effect in muscle, as this proteoglycan is known to modulate pro-angiogenic factors such as fibroblast growth factor 2 (FGF2), vascular endothelial growth factor (VEGF) and platelet-derived growth factor (PDGF) reviewed by Iozzo et al. [46].

## 4. Discussion

In this review, evidence from 21 studies including 402 participants was analysed to investigate the effect of exercise training on intramuscular ECM composition and whether these ECMs are involved in the events of muscle adaptation and remodelling. There was a widespread increase in the expression of ECM molecules in response to exercise training stimulus. Intramuscular ECMs were altered after training interventions at the transcriptional and translational level. All types of exercise training significantly upregulated similar numbers of collagens, glycoproteins, proteoglycans. Structural ECMs are likely to be widely increased supporting muscle remodelling. While it became evident that ECMs participate in muscle adaptation, the current evidence on the role of ECMs orchestrating particular events during remodelling was limited by lack of controlled trials incorporating mechanistic outcome measures into adequately sized human exercise training trials.

All studies demonstrated that repetitive bouts of exercise over time (training) promotes changes in muscle architecture, capillarisation and myogenesis in younger and older participants, consistent with other studies [4,13,17,54,58]. Despite the variability regarding participant sex, age and exercise type/duration, muscle remodelling was accompanied by ECM adaptation. This was mostly evident in seven studies, which associated ECM changes to specific muscle remodelling events including CSA, muscle strength, angiogenesis, as well as the level of physical activity [27,36,40,41,42,43,44].

Exercise is known to affect muscle in young and old adults differently. Our findings revealed that similar ECMs are increased in both age groups after exercise training. Only two studies compared the exercise training-induced ECM response in younger and older participants [36,38]. Their results were in agreement, showing that change in ECMs is towards the same direction in young and older participants. Although the fold change may be different among age groups and exercise training applied, the ECM responses are towards a similar direction. In agreement, it was observed that ECMs upregulated in early training sessions are also upregulated at later sessions. [36]. Thus, the findings indicate that in healthy adults, exercise training leads to similar ECM transcriptomes independently of age group and number of training sessions, demonstrating similar regulation in both age groups.

The ECM response is resultant of a force–time integral among the interventions tested, which may have affected the fold change of individual genes, but resulted in similar transcriptomes [59]. Further understanding of how different types of exercise cause changes in intramuscular ECMs was limited by the number of studies comparing exercise modalities [38], as AET and HIIT were mostly prescribed to younger populations and CT and RET to the older age groups. Likewise, rat skeletal muscle presented similarly increased ECM transcriptomes in response to isometric and concentric exercise training, but greater fold change was observed in response to higher mechanical stimulus [60]. Considering that the changes in gene expression after exercise in the elderly are delayed compared to younger participants [59], the tendency of applying longer training protocols to the elderly may have contributed to the observed similar ECM outcomes. In agreement with our findings, the study on acute RET bouts demonstrated that ECMs, including COL1A1, COL7A7, ADAMTS1, are upregulated in participants from both age groups [61]. Importantly, all exercise types upregulated collagen IV and laminin in both age groups. These ECMs provide critical scaffolding for the basement membrane, offering stability for the sarcolemma and myofiber cytoskeletal integrity, and the lateral transfer of mechanical force from the muscle cell to surrounding stroma ECM during contraction. The observed ECM remodelling at the basement membrane, in addition to facilitating myofiber repair, will allow the healthy expansion of intramuscular ECM to accommodate muscle fibre growth. Therefore, this systematic review demonstrates that adequate training interventions in terms of duration and intensity were applied according to age groups, and ECM adaptation accompanied muscle phenotypic remodelling [27,36,42,43].

Quiescent satellite cells which reside beneath the basal lamina can respond to mechanical stimuli and damage, becoming activated, differentiating into myoblasts that can fuse together to regenerate lost tissue or fuse with existing fibres to allow for myofiber repair [62]. This process depends on ECM composition, stiffness, topography and porosity [20]. The observed balanced composition of ECMs between age groups highlights the importance of ECMs’ function not only as a supportive scaffold, but also integrating both biochemical and mechanical signalling in the stem cell niche [63]. Therefore, ECMs play a role regulating cell behaviour, dictating tissue repair, remodelling and overall function [20]. At the early stages of muscle regeneration and repair, laminin and collagen type I are downregulated. In contrast, hyaluronic acid, tenascin and fibronectin are upregulated, forming a provisional matrix, enabling mitotic satellite cell adhesion, fusion and muscle regeneration [9].

Fibronectin, the preferred adhesive substrate for satellite cells, was reportedly increased only in young participants. Further studies on the effect of exercise training on the expression of fibronectin in the older population will be necessary to understand the importance of this glycoprotein to myogenesis. It is noteworthy that loss of muscle fibronectin in aged mice leads to ineffective muscle remodelling [64]. As satellite cells fail to adhere to fibronectin, the cells die by anoikis, demonstrating that ECM composition controls remodelling. The study also demonstrated that overexpression of fibronectin leads to rejuvenation of the skeletal muscle. In addition, fibronectin has been shown to prevent myostatin-mediated inhibition of myoblast proliferation [65,66]. Decorin is another ECM upregulated after exercise training, which is capable of inhibiting myostatin [27,34]. Myostatin regulates myogenesis and inhibits muscle mass by maintaining satellite cell quiescence. Recently, the group of Barreiro demonstrated that sarcopenic muscle from COPD patients is characterised by increased levels of myostatin compared to non-sarcopenic controls [58]. However, the levels of decorin and fibronectin in sarcopenic muscle after exercise training is not known.

Endothelial cell adhesion is mediated by the counterbalancing properties of fibronectin and tenascin-C [67]. The antiadhesive tenascin C, previously associated with muscle damage, is shown in this review to be crucial to increased capillarisation in young participants after AET postexercise training [40]. The authors concluded that the A/A genotype for tenascin C is more permissive of structural rearrangements of capillaries with respect to muscle fibres by relieving cells from the mechanical constraints of contact inhibition. For individuals carrying the T/T genotype, angiogenesis remained unchanged, suggesting that genotyping of tenascin C affects the exercise-induced level of capillarization [40].

Glypican-4 and chondroadherin are proteoglycans reported only in the older group and were among the few ECMs downregulated after exercise training [26]. The heparan sulphate proteoglycans, such as glypican-4, agrin and perlecan, are intimately associated with the plasma membrane of satellite cells functioning as major modifiers of growth factors such as FGF and VEGF. Glypican-4 also functions as an adipokine, which is associated with metabolic disease [68]. The downregulation of glypican-4 in an older group [26] is herein another supportive finding on the effect of ECM regulating muscle metabolism [68]. Increased levels of glypican-4 are associated with risk of type 2 diabetes and the prevalence of insulin resistance in these patients [69].

None of the studies compiled in this analysis included participants presenting muscle and nerve pathologies, sarcopenia, cachexia, dystrophic muscle repair, fibrosis or abnormal muscle remodelling. Nor were any damaging exercise interventions included. Thus, we can conclude that the that the observed widespread increase in ECMs reflects a physiological ECM response and adaptation to exercise training in all age groups tested.

### Strength and Limitations

To the best of our knowledge, this is the first systematic review on the effect of exercise training-induced ECM adaptations in human skeletal muscle. Only training exercise interventions which displayed a balanced insight of the post-training adaptations and repair of the muscle tissue were retained in this review. The effect of an acute bout of exercise was not the topic of this review, as that would reflect momentarily changes in ECM which may not be representative of a physiological muscle tissue adaptation, especially when considering the variability in human response. Importantly, only data from healthy adults were analysed, allowing direct translation of this research into understanding the benefits of exercise training improving muscle health. However, due to the different methodological approaches used in the studies, further data analyses were restricted. Future research on exercise training should be focused on addressing how the skeletal muscle ECM of a specific population group (e.g., sex and age) responds to a specific exercise training intervention. Deciphering the mechanisms that underlie ECM-induced skeletal muscle adaptation will support the discovery of biomarkers of ECM adaptation, which can present itself as a novel therapeutic target, contributing to the understanding of muscle remodelling myogenic processes and supporting the use of exercise training as medicine.

## 5. Conclusions

The importance of exercise training conditioning muscle has long been recognised for its therapeutic effect in healthy adults and counteracting muscle wasting [2,58]. Recently, advances have supported better understanding of the potential of exercise training for maintaining muscle mass, strength and function during ageing. Our findings demonstrate that exercise training affects ECM composition and, consequently, adaptation and regeneration after exercise. The intramuscular ECMs, consisting of collagens, proteoglycans and glycoproteins, have a well-defined role in three-dimensional scaffolding and are also essential for effective muscle contraction and force transmission. This systematic review also points to the involvement of ECMs in myogenic and angiogenic physiological processes, and it is clear that the ECM, by interacting with various cells, can regulate exercise-induced muscle adaptation, regeneration and repair. Further studies on the effect of exercise training modifying ECMs in different age groups are necessary to better understand the crucial role of these molecules on ageing muscle. New findings on the role of ECMs will support the development of effective therapeutic approaches to support and treat muscle wasting.

## Figures and Tables

**Table 1:** ROBINS-I quality assessment scores for the non-RCTs included studies.

Author	Bias Due to Confounding	Bias in Selection of Participants into the Study	Bias in Classification of Interventions	Bias Due to Deviations from Intended Interventions	Bias Due to Missing Data	Bias in Measurement of Outcomes	Bias in Selection of the Reported Result	Overall Bias
Damas et al., 2018 [24]	Low	Low	Low	Low	Low	Low	Low	Low
Deshmukh et al., 2021 [25]	Low	Low	Low	Low	Low	Moderate	Low	Moderate
Hjorth et al., 2015 [26]	Low	Moderate	Low	Low	Low	Unclear	Low	Moderate
Kanzleiter et al., 2014 [27]	Low	Low	Low	Low	Low	Unclear	Low	Moderate
Karlsen et al., 2020 [28]	Low	Low	Low	Low	Low	Moderate	Low	Moderate
Kern et al., 2014 [29]	Moderate	Low	Low	Low	Low	Low	Low	Moderate
Makhnovskii et al., 2020 [30]	Low	Moderate	Low	Unclear	Low	Low	Low	Moderate
Nishida et al., 2010 [31]	Low	Low	Low	Low	Low	Low	Low	Low
Norheim et al., 2011 [32]	Low	Unclear	Low	Low	Low	Low	Low	Moderate
Norheim et al., 2014 [33]	Low	Low	Low	Low	Low	Unclear	Low	Moderate
Olstad et al., 2020 [34]	Unclear	Low	Low	Low	Low	Low	Low	Moderate
Radom-Aizak et al., 2005 [35]	Low	Low	Low	Low	Low	Low	Unclear	Moderate
Raue et al., 2012 [36]	Low	Low	Low	Low	Low	Low	Low	Low
Riedl et al., 2010 [37]	Low	Low	Low	Low	Low	Low	Low	Low
Robinson et al., 2017 [38]	Low	Low	Low	Low	Low	Low	Low	Low
Timmons et al., 2010 [39]	Low	Low	Low	Low	Low	Low	Low	Low
Valdivierso et al., 2017 [40]	Low	Low	Low	Low	Low	Low	Low	Low
Walton et al., 2019 [41]	Low	Low	Low	Low	Moderate	Low	Low	Moderate

**Table 2:** RoB 2 quality assessment scores for RCTs included studies.

Author	Randomization Process Bias	Deviation from the Intended Intervention Bias	Missing Outcome Bias	Measurement of the Outcome Bias	Selection of Reported Results Bias	Overall Risk of Bias
Alghadir et al., 2016 [42]	High	Low	Low	High	Moderate	High
Fragala et al., 2014 [43]	Low	Low	Low	Unclear	Low	Moderate
Kim et al., 2015 [44]	Low	Low	Low	Unclear	Low	Moderate

**Table 3:** Characteristics of the included studies, their participants and muscle remodelling outcome.

First Author, Year of Publication	Country	Participant Group (n);	Age ± SD	Study Design	Experimental Group Intervention	Experimental Duration and Frequency,	Attrition	Outcome Measures	Δ Muscle Remodelling Post-Training (within Group)	Δ Muscle Remodelling Post-Training (between Groups)
		Sex (n, %)	or ± SEM(*)			Dosage (h)	(Reasons)			
Damas et al., 2018 [24]	Brazil	Exercise (9); Male (9, 100%)	26 ± 2	Pre–post study	RET: it involved two exercises for lower body.	10 weeks (2x/week) Dosage = N/A	^1^ participant (male) removed	N/A	N/A	N/A
Deshmukh et al., 2021 [25]	Denmark	Exercise (5); Male (5, 100%)	24 ± 1 *	Sub-cohort of pre–post study	AET: participants performed indoor cycling exercise (intensity ranged from 75–90% of maximal heart rate): 3 out of 4 sessions performed at home, 1 out of 4 at the laboratory.	12 weeks (4x/weeks) Dosage = 48	None	NA	NA	NA
Fragala et al., 2014 [43]	USA	Exercise (12); Male (7, 58.3%), Female (5, 41.7%)	70.5 ± 6.9	Pilot RCT	Supervised RET: 60–90 min (≈70–85% of RM).	6 weeks (2x/week) Dosage = 12–18	None	*Muscle strength/* *capacity*		
								Leg extension strength (kg)	Exercise: ↑ 29.0% (*p* < 0.001) Control: NS	NS

				
		Control (11); Male (6, 54.5%), Female (5, 45.5%)	69.6 ± 5.5		Maintenance of normal physical activities.			Muscle quality (relative strength)	Exercise: ↑ 28.0% (*p* < 0.001) Control: NS	NS

	
								*Body composition*		
								LBM (kg)	Exercise:↑ 0.2% (NS)	NS
									Control: NS	
								*Muscle architecture*		
								Muscle cross-sectional area (CSA) (cm^2^)	Exercise:↑ 8.3% (NS) Control: NS	NS

Hjorth et al., 2015 [26]	Norway	Exercise (26); Male (26, 100%)	51.2 ± 6.6	Pre–post study	Supervised CT: 2 intervals bicycle sessions and 2 whole body strength-training sessions per week. Each session lasted 1 h.	12 weeks (4x/week) Dosage = 48	None	*Muscle strength/* *capacity*		N/A
								Leg extension strength (kg)	↑ 9.6% (*p* < 0.001)	

								*Body composition*		
								Fat mass (L)	↓ 8.2% (*p* < 0.001)	
								*Muscle architecture*		
								Thigh muscle area (cm^2^)	↑ 7.5% (*p* < 0.001)	

								*Ventilatory changes*		
								VO_2max_ (mL/kg*min)	↑ 11.2% (*p* < 0.001)	
Kanzleiter et al., 2014 [27]	Norway/Germany	Exercise (26); Male (26, 100%); Normal glucose group (13); Pre-diabetes group (13)	51.2 ± 6.6	Pre–post study	Supervised CT: 2 intervals bicycle sessions and 2 whole body strength-training sessions per week. Each session lasted 1 h.	12 weeks (4x/week) Dosage = 48	None	N/A	N/A	N/A
Karlsen et al., 2020 [28]	Denmark			Pre–post study	Supervised heavy-load RET: sessions involved 3 exercises for lower body and 2 optional for upper body	13 weeks (3x/week) Dosage = N/A	^2^ Five old and two young participants did not complete the intervention.	*Muscle strength/* *capacity*		N/A
								Isometric knee extensor peak torque (Nm)	Young: ↑ 14.4 % (*p* < 0.01)	
									Older: ↑ 14.3% (*p* < 0.001)	
								Isokinetic knee extensor peak torque (Nm)	Young: ↑ 11.9% (*p* < 0.05)	
									Older: ↑ 9% (*p* < 0.05)	
								*Body composition*		
								Thin lean mass (kg)	Young: ↑ 6.7% (*p* < 0.001)	
		Young (7); Male (7, 100%)	25 ± 3						Older: ↑ 6% (*p* < 0.001)	
								*Muscle architecture*		
		Older (19); Male (19, 100%)	67 ± 4					CSA VL (μm^2^)	Young: ↑ 11.9% (*p* < 0.001)	

									Older: ↑ 14.5% (*p* < 0.001)	
								CSA QF (μm^2^)	Young: ↑ 8.9% (*p* < 0.01)	

									Older: ↑ 10.8% (*p* < 0.001)	
								CSA type I fibres (μm^2^)	Young: ↓0.2% (NS) Older: ↑ 5.7% (NS)	
								CSA type II fibres (μm^2^)	Young: ↑ 10.2% (NS)	
									Older: ↑ 25.8%	
									(*p* < 0.001)	
								Type I fibres (%)	Young: ↓ 2.3% (NS)	
									Older: ↑ 1.9% (NS)	
Kern et al., 2014 [29]	Italy/Austria		73.1 ± 6.9	Pre–post study	ES training at home: performed with a two-channel custom-built battery-powered stimulator3 × 10 min each session.	9 weeks (2x/week for the first 3 weeks and 3x/week for the following 6 weeks) Dosage = 12	None	*Muscle strength/* *capacity*		N/A
								Torque (Nm/kg)	↑ 6.0 ± 4.9 (*p* < 0.05)	
								*Muscle architecture*		
								All fibres size (μm)	NS	
		Exercise (16); Male (8, 50%), Female (8, 50%)						Type I fibres size (μm)	↓ 3.6% (*p* < 0.0001)	

								Type I fibres percentage (%)	↓ 7.2% (N.S)	
	
								Type IIa fibres size (μm)	↑ 2.2% (*p* < 0.0001)	

								Type IIa fibres percentage (%)	↑ 8.9% (N.S)	

Kim et al., 2015 [44]	Korea	Exercise (22); Female (22, 100%)	74.5 ± 0.6 *	RCT	RET: it involved 2 supervised and 3 home-based sessions. Progressive intensity of the intervention.	12 weeks (5x/week) Dosage = 60	^3^ Ten participants did not complete the intervention.	*Muscle strength/* *capacity*		
								Grip strength (kg)	Exercise:↑ 27.0% (*p* < 0.001)	*p* < 0.001
	
								Knee extensor strength 60°/s (N)	Exercise:↑ 42.1% (*p* < 0.001)	*p* = 0.019

								Knee flexor strength (N)	Exercise:↓ 1.5% (NS)	*p* = 0.002

		Control (8); Female (8, 100%)	76.05 ± 2.0 *		Maintenance of normal physical activities and performance of one hour stretching once a week			Knee extensor strength 180°/s (N)	Exercise:↑ 33.7% (NS)	NS

								Knee flexor strength (N)	Exercise:↓ 19.4% (*p* < 0.001)	*p* = 0.028
	
								*Body composition*		
								Waist–hip ratio (WHR)	Exercise:↓ 1.2% (NS)	N/A

								Arm circumference (cm)	Exercise:↓ 5.1% (NS)	N/A

								Thigh circumference (cm)	Exercise:↓ 1.3% (NS)	N/A

Makhnovskii et al., 2020 [30]	Russia	Exercise (7); Male (7, 100%)	22.5 ± 1.5 *	Pre–post study	AET: participants alternated continuous (intensity at 70% LT_4_) and intermittent exercise ((3 min, 50% LT_4_ + 2 min, 85% LT_4_) x12)) on different days.	5 weeks (7x/week) Dosage = 35	None	N/A	N/A	N/A
Nishida et al., 2010 [31]	Japan	Exercise (6); Male (6, 100%)	19–32	Pre–post study	Supervised AET: participants performed the session for 60 min using an upright cycle ergometer. Training intensity at the LT level.	12 weeks (5x/week) Dosage = 60	None	*Body composition*		N/A
								Fat percentage (%)	↓ 2.2% (NS)	
								*Ventilatory changes*		
								VO_2max_ (mL/kg * min)	↑ 8.7% (NS)	
								VO_2_ at LT (mL/kg * min)	↑ 62.5% (*p* < 0.05)	
								VO_2max_ at LT (%)	↑ 48.9% (*p* < 0.05)	
	
Norheim et al., 2011 [32]	Norway	Exercise (13); Male (13, 100%)	26.8 (19–35)	Sub-cohort of pre–post study	RET: it involved 1–3 sets of leg press, leg extension, leg curl, seated chest press, seated rowing, latissimus dorsi pull-down, biceps curl, and shoulder press.	11 weeks (3x/week) Dosage = N/A	None	N/A	N/A	N/A
Norheim et al., 2014 [33]	Norway	Exercise (26); Male (26, 100%), Normal glucose group (13), Pre-diabetes group (13)	51.2 ± 6.6	Pre–post study	Supervised CT: it involved 2 interval bicycle sessions and 2 whole body strength-training sessions per week. Each session lasted 1 h.	12 weeks (4x/week) Dosage = 48	None	N/A	N/A	N/A
Radom-Aizak et al., 2005 [35]	Israel	Exercise (6); Male (6, 100%)	68.0 ± 2.7 *	Pre–post study	AET: participants performed 45 min sessions (from the 3rd–12th week) on a cycle ergometer at 80% of the predetermined HR_max_.	12 weeks (3x/week) Dosage = 27	None	*Ventilatory changes*		N/A
								VO_2max_ (L/min)	↑ 17.8% (*p* = 0.009)	
								Anaerobic threshold (%)	↑ 21% (*p* = 0.008)	
	

	
	
	
Raue et al., 2012 [36]	USA	Young (16); Male (8, 50%), Female (8, 50%)	24 ± 4	Pre–post study	RET: it involved 3 sets of 10 bilateral knee extensions (70–75% of 1 RM).	12 weeks (3x/week) Dosage = N/A	None	*Muscle strength/* *capacity*		N/A

								Leg extension strength (kg)	↑ 5.7–↑ 41.3 kg	
		Older (12); Male (6, 50%), Female (6, 50%)	84 ± 3							
								*Muscle architecture*		
								Thigh muscle CSA (cm^2^)	↓ 1.2–↑ 10.4 cm^2^	
	
Riedl et al., 2010 [37]	Japan	Exercise (7); Male (7, 100%)	64 ± 2.6	Pre–post study	Supervised AET: participants performed sessions of 60 min on a cycle ergometer. Training intensity at the LT level	6 weeks (5x/week) Dosage = 30	None	*Body composition*		N/A
								Fat percentage (%)	↓ 9.9% (*p* < 0.05)	
								*Ventilatory changes*		
								VO_2_ at LT (%)	↑ 8.3% (*p* < 0.05)	
								VO_2_ max (mL/FFM kg/min)	↑ 7.3% (*p* < 0.05)	
	
Robinson et al., 2017 [38]	USA	Young HIIT exercise (10); Male, Female		Pre–post study	HIIT: participants performed 3 sessions per week of cycling (4 × 4 min at >90% of VO_2max_ separated by 3 min of pedalling at no load) and 2 sessions per week of treadmill walking (45 min at 70% of VO_2max_).	HIIT: 12 weeks (5x/week) Dosage = 34.8	^4^ Five young and three older participants did not complete the intervention. There was no information on number of males and females completing the study	*Young HIIT*		Absolute VO_2max_ (mL/min) in young: ↑ following HIIT (*p* < 0.0001) > ↑ following RET (*p* < 0.048) and CT (*p* = 0.0001)
			25.4 ± 4.3							
								*Ventilatory changes*		
								VO_2max_ (mL/kg BW/min)	↑ (*p* < 0.001)	
	
		Older HIIT exercise (8); Male, Female						*Body composition*		
			70.7 ± 4.6					FFM (kg)	↑ (*p* < 0.05)
								*Muscle strength/* *capacity*		Absolute VO_2max_ (mL/min) in older: ↑ following HIIT (*p* < 0.0091) and CT (*p* = 0.0096) >↑ following RET (ns)
	
		Young RET (10); Male, Female	23.7 ± 3.5					Maximal leg strength (1 RM) leg press (AU/kg Leg FFM)	↑ (NS)	
			
					RET: participants performed 2 sessions of lower and upper body exercises (4 sets of 8–12 repetitions), 2 days each per week.	RET: 12 weeks (5x/week) Dosage = N/A				
			
		Older RET (8); Female	70.3 ± 3.9							
								*Older HIIT*		
								*Ventilatory changes*		Relative VO_2max_ (mL/min) in young: ↑ ~28% following HIIT (*p* < 0.0001) > ↑ ~17% following CT (*p* < 0.0001) > RET (ns)
		Young Combined exercise (8); Male, Female	26.3 ± 2.7					VO_2max_ (mL/kg BW/min)	↑ (*p* < 0.01)	
		
					CT after a 3 months SED: Following SED, participants underwent metabolic studies and performed CT of 5 days per week cycling (30 min at 70% VO_2max_) and 4 days per week weightlifting with fewer repetitions than RET.	Combined:12 weeks (9x/week) Dosage > 30		*Body composition*		
								FFM (kg)	↑ (*p* < 0.05)	
								*Muscle strength/* *capacity*		
		Older Combined exercise (7); Male, Female	68.6 ± 3.4							
	
								Maximal leg strength (1 RM) leg press) (AU/kg Leg FFM	↑ (NS)	Relative VO_2max_ (mL/min) in older: ↑ ~21% following CT (*p* < 0.0001) > ↑ ~17% following HIIT (*p* < 0.0001) > RET (ns)
	

	
								*Young RET*		
								*Ventilatory changes*		
								VO_2max_ (mL/kg	↑ (NS)	
								BW/min)		
								*Body composition*		Leg strength: ↑ Following RET and CT > HIIT (NS)
								FFM (kg)	↑ 4% (*p* < 0.0001)	
	
								*Muscle strength/*		
								Maximal leg strength	↑ (*p* < 0.05)↑	
								(1 RM) leg press		
								(AU/kg Leg FFM)		
	
								*Older RET*		
								*Ventilatory changes*		
								VO_2max_ (mL/kg BW/min)	↑ (NS)	

								*Body composition*		
								FFM (kg)	↑ (*p* < 0.01)	
								*Muscle strength/* *capacity*		

								Maximal leg strength (1 RM) leg press (AU/kg Leg FFM)	↑ (*p* < 0.05)	


	
								*Young CT*		
								*Ventilatory changes*		
								VO_2max_ (mL/kg BW/min)	↑ (*p* < 0.001)	

								*Body composition*		
								FFM (kg)	↑ (*p* < 0.05)	
								*Muscle strength/* *capacity*		
								Maximal leg strength (1 RM) leg press (AU/kg Leg FFM)	↑ (*p* < 0.05)	


	
								*Older CT*		
								*Ventilatory changes*		
								VO_2max_ (mL/kg BW/min)	↑ (*p* < 0.01)	

								*Body composition*		
								FFM (kg)	↑ (*p* < 0.05)	
								*Muscle strength/* *capacity*		
								Maximal leg strength (1 RM) leg press (AU/kg Leg FFM)	↑ (*p* < 0.05)	


Timmons et al., 2010 [39]	Sweden, Denmark, UK, USA	Exercise (24); Male (24, 100%)	23	Pre–post study	Supervised AET: participants performed 45 min cycling sessions. Training intensity customized to 70% of the pretraining VO_2max_.	6 weeks (4x/week) Dosage = 18	None	*Ventilatory changes*		N/A
								VO2 max (L/min)	↑ 14% (N/A)	
								Submax RER (ratio)	↓ 10% (N/A)	
	
	
	
Valdivierso et al., 2017 [40]	Switzerland	Exercise (61); Male (61, 100%), A/A alleles (12), A/T alleles (38), T/T alleles (11)	29.5 ± 9.3	Pre–post study	AET: participants performed 30 min sessions on a cycle ergometer at a heart rate corresponding to 65% of Pmax. Training intensity maintained at ≈90% of maximal heart rate	6 weeks (5x/week) Dosage = 15	None	*Muscle architecture*		
								Muscle fibre area (μm^2^)	↑ 8.3% (NS)	

								Biopsy myofibrils (%)	↓ 4.0% (*p* < 0.05)	

								Capillary-to-fibre ratio (ALL)	↑ 12.1% (*p* < 0.05)	

								Capillary-to-fibre ratio:		
								A/A genotype	↑ 25.0% (NS)	A/A vs. T/T (*p* < 0.05)
								A/T genotype	↑ 12.6% (*p* < 0.05)	A allele carriers vs. T/T (*p* < 0.05)
								T/T genotype	↓ 12.5% (NS)	
				
								Capillary density (mm^−2^)	↓ 5.5% (*p* < 0.05)	

								*Ventilatory changes*		
								VO_2_max (mL/kg * min)	↑ 8.5% (*p* < 0.05)	

								Pmax (ergospirometry) (W)	↑ 12.7% (*p* < 0.05)	

Walton et al., 2019 [41]	USA	Exercise (20); Male (4, 25%), Female (16, 75%)	49.8 ± 2.3 *	Pre–post study	AET: participants performed 45 min sessions using a stationary cycle ergometer (at a target intensity corresponding to 65% of VO_2max_ and ≈75–80% of maximum heart rate)	12 weeks (3x/week) Dosage = 27	None	N/A	N/A	N/A
Alghadir et al., 2016 [42]	Saudi Arabia	Exercise (25); Male with T2D (25, 100%)	48.8 ± 14.6	RCT	Supervised AET: 50 min in intensity defined by heart rate (THR max; 60–70%)	12 weeks (3x/week) Dosage = 30	None	N/A	N/A	N/A
		Control (25); Male (25, 100%)	48.7 ± 3.4		Sedentary lifestyle					
		
Olstad et al., 2020 [34]	Norway			Pre–post study	Supervised heavy-load RET: it involved all major muscle groups. Gradual progression on the training loads was applied. Each session lasted ≈60 min.	13 weeks (3x/week) Dosage = 39	^5^ One participant did not complete the study	*Muscle strength/* *capacity*		N/A
		Exercise healthy (18); Female (18, 100%)	73.9 ± 5.7							
								Relative strength	Healthy: ↑ 32 ± 16%	
									Osteoporotic: ↑ 31 ± 19%	
		Exercise osteoporotic (17); Female (17, 100%)	78.0 ± 6.2							


The last two entries are from clinical populations. Data are presented as mean ± SD or ± SEM (*); number (n); hours (h); randomised controlled trial (RCT); resistance exercise training (RET); combined training (CT); aerobic exercise training (AET); high intensity interval training (HIIT); sedentary period (SED); electrical stimulation (ES); decrease (↓); not significant (NS); not available (N/A); repetition maximum (RM); lean body mass (LBM); cross sectional area (CSA); increase (↑); maximum training heart rate (max THR); maximal aerobic capacity (VO_2max_); maximal accumulated oxygen deficit (MAOD); voluntary repetition maximum (VRM); submaximal exercise respiratory exchange ratio (Submax RER); lactate threshold (LT); lactate threshold at 4 mmol/l (LT_4_); body weight (BW); fat free mass (FFM); absolute units (AU); intravenous failure (IV failure); Type 2 Diabetes (T2D); vastus lateralis (VL); quadriceps femoris (QF). Attrition: ^1^ A male participant was removed from analysis because of poor sample quality. ^2^ Dropouts and medical issues kept five older and two young participants out of the final analysis. ^3^ Ten subjects (four in the exercise group, six in the control group) could not complete the study. Reasons: difficulties of time commitment and loss of motivation. ^4^ Five young adults dropped out from the study. Reasons: (a) time constraints (n = 2), (b) medical unrelated to the study (n = 2), (c) and IV failure (n = 1). Three older adults dropped out. Reasons: (a) medical unrelated to the study (n = 1), (b) did not want to perform follow up testing (n = 1), and (c) completed sedentary-only portion (n = 1). ^5^ A compression fracture in the spine was attained during an accident in the squat exercise. Recovery of the patients was succeeded after 3 months of reduced loading.

**Table 4:** Exercise training changes ECMs at protein and mRNA level and are associated with muscle remodelling outcome.

First Author, Year of Publication	Outcome Measure	Δ ECM Outcome Post Training within Group	Association between ECM and Muscle Remodelling Outcome within the Study
Damas et al., 2018 [24]	*mRNA expression*		N/A
	*Collagens*		
	COL3A1	↑ 146% (*p* < 0.05)	
	COL4A1	↑ 112% (*p* < 0.05)	
	COL5A2	↑ 95% (*p* < 0.05)	
	*Glycoproteins*		
	CTHRC1	↑ 105% (*p* < 0.05)	
	LAMB1	↑ 79% (*p* < 0.05)	
	THBS4	↑ 144% (*p* < 0.05)	
	PXDN	↑ 81% (*p* < 0.05)	
Deshmukh et al., 2021 [25]	*Protein expression*		N/A
	*Glycoproteins*		
	Agrin	Slow fibres: ↑ 147% (NS)	
		Fast fibres: ↑ 460% (*p* < 0.001)	
		Whole muscle: ↑ 130% (NS)	
	
	Thrombospondin 4	Slow fibres: ↑ 240% (NS)	
		Fast fibres: ↑ 360% (*p* = 0.015)	
		Whole muscle: N/A	
	Peroxidasin	Slow fibres: ↑ 320% (*p* = 0.0017)	
		Fast fibres: ↑ 300% (NS)	
		Whole muscle: ↑ 360% (*p* = 0.0018)	
	Dermatopontin	Slow fibres: ↓ 70% (NS)	
		Fast fibres: ↓ 85% (NS)	
		Whole muscle: ↓ 16% (*p* = 0.0046)	
	Fibrillin-1	Slow fibres: ↓ 100% (NS)	
		Fast fibres: ↓ 82% (NS)	
		Whole muscle: ↑ 280% (*p* = 0.0063)	
	Irisin precursor, fibronectin type III	Slow fibres: ↑ 108% (NS)	
		Fast fibres: ↓ 90% (NS)	
		Whole muscle: ↓ 70% (*p* = 0.025)	
	IGFN1 (Immunoglobulin-like and fibronectin type III domain containing	Slow fibres: ↑ 790% (*p* = 0.0028)	
		Fast fibres: ↑ 560% (*p* = 0.011)	
		Whole muscle: ↑ 225% (NS)	
	Laminin subunit alpha-1	Slow fibres: N/A	
		Fast fibres: N/A	
		Whole muscle: ↑ 370% (*p* = 0.0415)	
	Laminin subunit alpha 4	Slow fibres: ↑ 160% (*p* = 0.0119)	
		Fast fibres: ↑ 143% (NS)	
		Whole muscle: ↓ 93% (NS)	
	Laminin subunit alpha 5	Slow fibres: ↑ 119% (NS)	
		Fast fibres: ↑ 99% (NS)	
		Whole muscle: ↑ 60% (*p* = 0.0431)	
	Microfibrillar-associated protein 4	Slow fibres: ↓ 90% (NS)	
		Fast fibres: ↓ 39% (*p* = 0.043)	
		Whole muscle: ↑ 137% (NS)	
	Microfibrillar-associated protein 5	Slow fibres: ↑ 440% (*p* = 0.0197)	
		Fast fibres: ↑ 62% (NS)	
		Whole muscle: ↑ 162% (NS)	
	Picachurin (EGFLAM)	Slow fibres: ↑ 300% (*p* = 0.0197)	
		Fast fibres: ↑ 510% (*p* < 0.001)	
		Whole muscle: ↑ 330% (*p* = 0.0008)	
Fragala et al., 2014 [43]	*Circulating markers (serum)*		P3NP vs. LBM (r = 0.422, *p* = 0.045)
	C-terminal agrin fragment (CAF)	Exercise: ↑ 10.4% (NS)	
		Control: ↑ 0.3% (NS)	CAF vs. VL CSA (r = 0.542, *p* = 0.008)
		
	N-terminal peptide of procollagen type III (P3NP)	Exercise: ↑ 7.9% (NS)	P3NP vs. muscle strength/quality (NS)
		Control: ↑ 1.9% (NS)	
			CAF vs. muscle strength/quality (NS)
Hjorth et al., 2015 [26]	*mRNA expression*		N/A
	*Collagens*		
	COL1A1	↑ 140% (*p* < 0.001)	
	COL1A2	↑ 80% (*p* < 0.001)	
	COL3A1	↑ 140% (*p* < 0.001)	
	COL4A1	↑ 140% (*p* < 0.001)	
	COL4A2	↑ 120% (*p* < 0.001)	
	COL5A1	↑ 50% (*p* < 0.001)	
	COL5A2	↑ 60% (*p* < 0.001)	
	COL6A6	↑ 100% (*p* < 0.001)	
	COL14A1	↑ 80% (*p* < 0.001)	
	COL15A1	↑ 50% (*p* < 0.001)	
	COL18A1	↑ 50% (p < 0.001)	
	*Proteoglycans*		
	ASPN	↑ 80% (*p* < 0.001)	
	BGN	↑ 110% (*p* < 0.001)	
	HSPG2	↑ 50% (*p* < 0.001)	
	OGN	↑ 110% (*p* < 0.001)	
	OMD	↑ 80% (*p* < 0.001)	
	ECM2	↑ 60% (*p* < 0.001)	
	LUM	↑ 50% (*p* < 0.001)	
	GPC4	↓ N/A (*p* < 0.001)	
	CHAD	↓ 52% (*p* < 0.001)	
	CSPG4	↑ 50% (*p* < 0.001)	
	*Glycoproteins*		
	AGRN	↑ 60% (*p* < 0.001)	
	LAMA4	↑ 70% (*p* < 0.001)	
	LAMB1	↑ 70 % (*p* < 0.001	
	LAMB3	↓ 56% (*p* < 0.001)	
	LAMC3	↑ 70% (*p* < 0.001)	
	THBS1	↑ 60% (*p* < 0.001)	
	THBS4	↑ 220% (*p* < 0.001)	
	NID1	↑ 60% (*p* < 0.001)	
	NID2	↑ 70% (*p* < 0.001)	
	PXDN	↑ 200% (*p* < 0.001)	
	ELN	↑ 50% (*p* < 0.001)	
	EMILIN3	↑ 60% (*p* < 0.001)	
	SPARC	↑ 80% (*p* < 0.001)	
	CTHRC1	↑ 70% (*p* < 0.001)	
Kanzleiter et al., 2014 [27]	*mRNA expression*		
	*Proteoglycans*		Δ Decorin expression vs. ΔLeg
	DCN	Healthy: ↑ (*p* < 0.05)	press strength (kg) (r = 0.56, *p* = 0.047)
		Pre-diabetes: ↓ (NS)	
Karlsen et al., 2020 [28]	*mRNA expression*		
	*Collagens*		
	COL1A1	Young: (NS)	
		Older: ↑ (values not reported) (*p* < 0.05)	
Kern et al., 2014 [29]	*mRNA expression*		
	*Collagens*		
	COL1	↑ (*p* < 0.005)	
	COL3	↑ (*p* < 0.005)	
	COL6	↑ (*p* < 0.005)	
Kim et al., 2015 [44]	*Circulating markers (serum)*		
	Irisin	Exercise: ↑ 22.5% (*p* < 0.05)	Irisin vs. grip strength
		Control: NS	(r = 0.526, p = 0.002)
		
			Irisin vs. leg strength
			(r = 0.414, *p* = 0.003)
Makhnovskii et al., 2020 [30]	*mRNA expression*		N/A
	*Collagens*		
	COL1A1	↑ 1060% (*p* < 0.05)	
	COL1A2	↑ 440% (*p* < 0.05)	
	COL3A1	↑ 656% (*p* < 0.05)	
	COL4A2	↑ 439% (*p* < 0.05)	
	COL6A1	↑ 196% (*p* < 0.05)	
	COL6A2	↑ 232% (*p* < 0.05)	
	COL6A3	↑ 273% (*p* < 0.05)	
	COL14A1	↑ 608% (*p* < 0.05)	
	COL15A1	↑ 271% (*p* < 0.05)	
	*Proteoglycans*		
	ASPN	↑ 359% (*p* < 0.05)	
	BGN	↑ 435% (*p* < 0.05)	
	HSPG2	↑ 204% (*p* < 0.05)	
	OGN	↑ 421% (*p* < 0.05)	
	LUM	↑ 447% (*p* < 0.05)	
	DCN	↑ (NS)	
	PRELP	↑ (NS)	
	*Glycoproteins*		
	LAMB1	↑ 326% (*p* < 0.05)	
	LAMC1	↑ 175% (*p* < 0.05)	
	*Protein expression*		
	*Collagens*		
	Collagen Type I Alpha 1 Chain	↑ 171% (*p* < 0.05)	
	Collagen Type I Alpha 2 Chain	↑ 173% (*p* < 0.05)	
	Collagen Type III Alpha 1 Chain	↑ 221% (*p* < 0.05)	
	Collagen Type XIV Alpha 1 Chain	↑ 164% (*p* < 0.05)	
	Collagen Type VI Alpha 1 Chain	↑124% (*p* < 0.05)	
	Collagen Type VI Alpha 2 Chain	↑121% (*p* < 0.05)	
	Collagen Type VI Alpha 3 Chain	↑124% (*p* < 0.05)	
	*Proteoglycans*		
	Asporin	↑ 152% (*p* < 0.05)	
	Lumican	↑ 123% (*p* < 0.05)	
	Prolargin (or Proline and arginine rich end leucine rich repeat protein)	↑ 118% (*p* < 0.05)	
Nishida et al., 2010 [31]	*mRNA expression (using SAGE)*		N/A
	*Collagens*		
	COL1A2	↑ 1200% (*p* < 0.05)	
	*Proteoglycan*		
	DCN	↓ 2100% (*p* < 0.05)	
Norheim et al., 2011 [32]	*mRNA expression*		N/A
	*Collagens*		
	COL1A1	M. VL: ↑ 520 (*p* < 0.05)	
		M. TRAP: ↑ 4340% (*p* < 0.05)	
	*Proteoglycans*		
	LUM	M. VL: ↑ 250 (*p* < 0.05)	
		M. TRAP: ↑ 430 (*p* < 0.05)	
	ECM1	M. VL: ↑ 180 (*p* < 0.05)	
		M. TRAP: ↑ 190 (*p* < 0.05)	
	*Glycoproteins*		
	SPARC	M. VL: ↑ 290 (*p* < 0.05)	
		M. TRAP: ↑ 960 (*p* < 0.05)	
	FN1	M. VL: ↑ 180 (*p* < 0.05)	
		M. TRAP: ↑ 250 (*p* < 0.05)	
Norheim et al., 2014 [33]	*mRNA expression*		N/A
	FNDC5 (Irisin)	Healthy: ↑ 40% (*p* < 0.05)	
		Pre-diabetes: ↑ 100% (*p* < 0.01)	
	*Circulating markers (serum)*	Healthy: ↓ (NS)	
	Irisin	Pre-diabetes: ↓ (NS)	
Radom-Aizak et al., 2005 [35]	*mRNA expression*		N/A
	*Collagens*		
	COL3A1	↑ 111% (*p* = 0.0178)	
Raue et al., 2012 [36]	*mRNA expression*		Pooled mRNA expression of COL4α2 vs. 1-RM (r = −0.418)
	*Collagens*		
	COL1A1	Young: N/A	Pooled mRNA expression of COL4α3 vs. 1-RM (r = −0.344-(−0.486))
		Older: ↑ 220–300%	
	COL1A2	Young: N/A	Pooled mRNA expression of COL4α4 vs. 1-RM (r = -0.540-(0.623))
		Older: ↑ 150–200%	Pooled mRNA expression of COL4α5 vs. 1-RM (r = −0.547)
	COL3A1	Young: N/A	Pooled mRNA expression of COLQ vs. 1-RM (r = 0.652)
		Older: ↑ 260–210%	Pooled mRNA expression of COL27α1 vs. 1-RM (r = −0.355-(−0.457))
	COL4A1	Young: ↑ 190–200%	Pooled mRNA expression of COL28α1 vs. 1-RM (r = −0.180)
		Older: N/A	Pooled mRNA expression of CSPG4 vs. 1-RM (r = 0.421)
	COL4A2	Young: ↑ 170%	Pooled mRNA expression of COL4α2 vs. CSA (r = −0.405)
		Older: ↑ 220%	Pooled mRNA expression of COL4α3 vs. CSA (r = −0.345-(−0.462))
	COL5A1	Young: N/A	Pooled mRNA expression of COL4α4 vs. CSA (r = −0.461-(0.486))
		Older: ↑ 290%	Pooled mRNA expression of COL4α5 vs. CSA (r = −0.406)
	COL5A2	Young: N/A	Pooled mRNA expression of COLQ vs. CSA (r= 0.540)
		Older: ↑ 180%	Pooled mRNA expression of COL27α1 vs. CSA (r = −0.348)
	COL5A3	Young: N/A	
		Older: ↑ 170–180%	
	COL15A1	Young: N/A	
		Older: ↑ 150%	
	*Proteoglycans*		
	ASPN	Young: N/A	
		Older: ↑ 200%	
	*Glycoproteins*		
	LAMA4	Young: N/A	
		Older: ↑ 174%	
	LAMB1	Young: N/A	
		Older: ↑ 165%	
	NID1	Young: N/A	
		Older: ↑ 160–200%	
	NID2	Young: N/A	
		Older: ↑ 194%	
	SPARC	Young: N/A	
		Older: ↑ 150–160%	
	THBS4	Young: N/A	
		Older: ↑ 168%	
	CTHRC1	Young: N/A	
		Older: ↑ 200%	
Riedl et al., 2010 [37]	*Number of tags per 100,000 SAGE tags*		
	*Collagens*		
	COL3A1	↑ 14	
	COL4A1	↑ 15	
	*Glycoproteins*		
	SPARC	↑ 20	
Robinson et al., 2017 [38]	*mRNA expression*		N/A
	*Collagens*		
	COL4A1	HIIT young: ↑ 217% (*p* ≤ 0.05)	
		HIIT older: ↑ 361% (*p* ≤ 0.05)	
		RET young: ↑ 267% (*p* ≤ 0.05)	
		RET older: ↑ 236% (*p* ≤ 0.05)	
		CT young: ↑ 185% (*p* ≤ 0.05)	
		CT older: ↑ 197% (*p* ≤ 0.05)	
	COL4A2	HIIT young: ↑ 188% (*p* ≤ 0.05)	
		HIIT older: ↑ 303% (*p* ≤ 0.05)	
		RET young: ↑ 219% (*p* ≤ 0.05)	
		RET older: ↑ 202% (*p* ≤ 0.05)	
		CT young: ↑ 172% (*p* ≤ 0.05)	
		CT older: ↑ 182% (*p* ≤ 0.05)	
	COL14A1	HIIT young: (NS)	
		HIIT older: ↑ 165% (*p* ≤ 0.05)	
		RET young: (NS)	
		RET older: (NS)	
		CT young: (NS)	
		CT older: (NS)	
	*Proteoglycans*	HIIT young: ↑ 174% (*p* ≤ 0.05)	
	ASPN	HIIT older: ↑ 232% (*p* ≤ 0.05)	
		RET young: ↑ 187% (*p* ≤ 0.05)	
		RET older: ↑ 243% (*p* ≤ 0.05)	
		CT young: ↑ 158% (*p* ≤ 0.05)	
		CT older: ↑ 177% (*p* ≤ 0.05)	
	LUM	HIIT young: (NS)	
		HIIT older: ↑ 156% (*p* ≤ 0.05)	
		RET young: (NS)	
		RET older: (NS)	
		CT young: (NS)	
		CT older: (NS)	
	ECM2	HIIT young: ↑ 175% (*p* ≤ 0.05)	
		HIIT older: ↑ 180% (*p* ≤ 0.05)	
		RET young: ↑161% (*p* ≤ 0.05)	
		RET older: ↑173% (*p* ≤ 0.05)	
		CT young: (NS)	
		CT older: (NS)	
	*Glycoproteins*		
	LAMB1	HIIT young: ↑ 171% (*p* ≤ 0.05)	
		HIIT older: ↑ 170% (*p* ≤ 0.05)	
		RET young: ↑ 154% (*p* ≤ 0.05)	
		RET older: (NS)	
		CT young: (NS)	
		CT older: ↑ 160% (*p* ≤ 0.05)	
	NID1	HIIT young: ↑ 160% (*p* ≤ 0.05)	
		HIIT older: ↑ 205% (*p* ≤ 0.05)	
		RET young: ↑152% (*p* ≤ 0.05)	
		RET older: ↑157% (*p* ≤ 0.05)	
		CT young: ↑155% (*p* ≤ 0.05)	
		CT older: ↑156% (*p* ≤ 0.05)	
	PXDN	HIIT young: ↑ 196% (*p* ≤ 0.05)	
		HIIT older: ↑266% (*p* ≤ 0.05)	
		RET young: ↑234% (*p* ≤ 0.05)	
		RET older: ↑209% (*p* ≤ 0.05)	
		CT young: ↑162% (*p* ≤ 0.05)	
		CT older: ↑166% (*p* ≤ 0.05)	
	SPARC	HIIT young: ↑ 188% (*p* ≤ 0.05)	
		HIIT older: ↑224% (*p* ≤ 0.05)	
		RET young: ↑170% (*p* ≤ 0.05)	
		RET older: ↑179% (*p* ≤ 0.05)	
		CT young: (NS)	
		CT older: ↑165% (*p* ≤ 0.05)	
	ELN	HIIT young: (NS)	
		HIIT older: ↑181% (*p* ≤ 0.05)	
		RET young: (NS)	
		RET older: (NS)	
		CT young: (NS)	
		CT older: (NS)	
	POSTN	HIIT young: (NS)	
		HIIT older: ↑ 167% (*p* ≤ 0.05)	
		RET young: (NS)	
		RET older: (NS)	
		CT young: (NS)	
		CT older: (NS)	
Timmons et al., 2010 [39]	*mRNA expression*		N/A
	*Collagens*		
	COL1A1	↑ 370–510%	
	COL1A2	↑ 90–550%	
	COL3A1	↑ 80–540%	
	COL4A1	↑ 350–430%	
	COL4A2	↑ 280–350%	
	COL5A1	↑ 240–270%	
	COL5A2	↑ 250–290%	
	COL5A3	↑ 50%	
	COL6A1	↑ 70%	
	COL6A2	↑ 210%	
	COL6A3	↑ 230%	
	COL8A1	↑ 260%	
	COL12A1	↑ 50%	
	COL14A1	↑ 250%	
	COL15A1	↑ 80%	
	COL18A1	↑ 60%	
	PLOD2	↑ 60%	
	*Proteoglycans*		
	ASPN	↑ 220–290%	
	BGN	↑ 250–560%	
	CSPG2	↑ 170–590%	
	HSPG2	↑ 90%	
	LUM	↑ 270%	
	OGN	↑ 420–500%	
	*Glycoproteins*		
	AGRN	↑ 60–80%	
	LAMA4	↑ 240%	
	LAMB1	↑ 250–270%	
	LAMC1	↑ 50%	
	SPARC	↑ 50–250%	
	NID1	↑ 80%	
	NID2	↑ 290%	
	FBN1	↑ 50–730%	
	FN1	↑ 60–90%	
	TNC	↑ 430–530%	
	THBS4	↑ 280%	
	POSTN	↑ 390%	
	PXDN	↑ 220–250%	
	FNDC1	↑ 300%	
	CTHRC1	↑ 340%	
Valdivierso et al., 2017 [40]	*Protein expression*		
	*Glycoproteins*		
	Tenascin C	A/A alleles: ↑ 138% (*p* < 0.05)	Capillary/fibre ↑
		A/T alleles: ↑ 77% (*p* < 0.05)	Capillary/fibre ↑
		T/T alleles: (NS)	Capillary/fibre ↓ 15%
Walton et al., 2019 [41]	*mRNA expression*		M2 macrophages/fibre vs. COL5A1 expression (r = 0.56, *p* = 0.021)
	*Collagens*		M2 macrophages/fibre vs. COL6A1 expression (r = 0.54, *p* = 0.026)
	COL5A1	↑ 53.6% (*p* = 0.013)	M2 macrophages/fibre vs. SPARC expression (r = 0.63, *p* = 0.007)
	COL6A1	↑ 29.5% (*p* = 0.009)	M2 macrophages/fibre vs. MMP14 expression (r = 0.69, *p* = 0.002)
	*Glycoproteins*		M2 macrophages/fibre vs. TGFβ1 expression (r = 0.50, *p* = 0.04)
	SPARC	↑ 56.6% (*p* < 0.001)	
Alghadir et al., 2016 [42]	*Circulating markers (serum)*		Level of cFN vs. Physical activity (PA):
	Cellular Fibronectin (or cFN)	Exercise: ↓ 53% (*p* < 0.001)	↑ in cFN after in low PA (r = 0.18, *p* < 0.001)
		Control: ↓ 2.1% (NS)	↑ in cFN after moderate PA (r = 0.12, *p* < 0.001)
			↑ in cFN after ↑ in high PA vs. (r = 0.14, *p* < 0.001)
Olstad et al., 2020 [34]	*mRNA expression*		N/A
	*Proteoglycans*		
	DCN	Healthy: N/A	
		Osteoporotic: ↑ 129.4%	
	*Glycoproteins*		
	SPARC	Healthy: N/A	
		Osteoporotic: ↑ 141.6%	
	MGP	Healthy: N/A	
		Osteoporotic: ↑ 128.2%	

The last two entries are from clinical populations. Data are presented as mean ± SD (or SEM *); lean body mass (LBM); cross sectional area (CSA); increase (↑); decrease (↓); not significant (NS); not available (N/A); change (Δ); serial analysis of gene expression tags (SAGE tags); resistance exercise training (RET); combined training (CT); high intensity interval training (HIIT); vastus lateralis muscle (MVL); trapezius muscle (MTRAP).

## Data Availability

Data used in this study were collated from peer reviewed studies. All databases were accessed via the Northumbria University library platform.

## Supplementary Materials

The systematic review search strategy for PUBMED (Table S1), and results on the changes in ECM-related molecules after exercise training intervention (Table S2) are available online at https://www.mdpi.com/article/10.3390/cells10051022/s1.
