# Supplementary material for: Exercise Training-Induced Extracellular Matrix Protein Adaptation in Locomotor Muscles: A Systematic Review
Source: Cells. 2021 Apr 26;10(5):1022. doi: 10.3390/cells10051022 (PMC8146973; doi:10.3390/cells10051022)
Supplement: Supplementary file 1 [file cells-10-01022-s001.zip › cells-1184465-supplementary.pdf]

Table S1. Systematic review search strategy for PUBMED

Category	Number	Search Term
**Population**	#1	Muscle disorder* [Mesh]
	#2	Cachexia [Mesh]
	#3	Sarcopenia [Mesh]
	#4	COPD [Mesh]
	#5	Muscle atrophy* [Mesh]
	#6	Aging [Mesh]
	#7	Skeletal muscle disease* [Text word]
	#8	Healthy aging [Text word]
	#9	Skeletal muscle dysfunction [Text word]
	#10	Muscle wasting [Text word]
	**#11**	**#1-10 (OR)**
**Intervention**	#12	Exercise [Mesh]
	#13	Physical activit* [Mesh]
	#14	Aerobic exercise* [Mesh]
	#15	Resistance training [Mesh]
	#16	Physical exertion [Mesh]
	#17	Inactivity [Text word]
	#18	HIIT [Text Word]
	#19	Acute bout [Text Word]
	#20	Repeated bout [Text word]
	#21	Endurance training [Text word]
	#22	Mechanical loading [Text word]

	**#23**	**#12-22 (OR)**

**Outcome**	#24	Extracellular matrix proteins [Mesh]
	#25	Extracellular matrix [Mesh]
	#26	Myogenic satellite cells [Mesh]
	#27	Skeletal muscle regeneration [Text Word]
	#28	Skeletal muscle remodel* [Text Word]
	#29	Extracellular matrix remodel* [Text Word]
	**#30**	**#24-29 (OR)**
	#31	Legs
	#32	Muscle, skeletal [Mesh]
	#33	Muscle, quadriceps [Text Word]
	#34	Peripheral muscle*[Text Word]
	#35	Locomotor muscle* [Text Word]
	**#36**	**#31-35 (OR)**
	**#37**	**30 AND 36**

Table S2. Changes in ECM-related molecules after exercise training intervention.

First author, year of publication	Outcome measure	Δ ECM outcome within group (pre-to-post exercise)
Damas *et al.*, 2018 [24]	*mRNA expression*	
	Miscellaneous	
	COLEC12	↑ 82% ($p < 0.05$)
	MXRA5	↑ 188% ($p < 0.05$)
Hjorth *et al.*, 2015 [26]	*mRNA expression*	
	Miscellaneous	
	ADAMTS7	↑ 80% ($p < 0.001$)
	ADAMTS15	↑ 60% ($p < 0.001$)
	ADAMTS8	↑ 60% ($p < 0.001$)
	ADAMTSL3	↑ 60% ($p < 0.001$)
	ADAMTS2	↑ 60% ($p < 0.001$)
	SERPINE1	↑ 60% ($p < 0.001$)
	SERPINH1	↑ 50% ($p < 0.001$)
	MMP14	↑ 50% ($p < 0.001$)
	IGFBP2	↑ 60% ($p < 0.001$)
	IGFBP3	↑ 70% ($p < 0.001$)
	KCP	↑ 70% ($p < 0.001$)
	WISP1	↑ 90% ($p < 0.001$)
	MXRA5	↑ 280% ($p < 0.001$)
	CLEC10A	↑ 70% ($p < 0.001$)
	LOXL2	↑ 220% ($p < 0.001$)
	LOX	↑ 200% ($p < 0.001$)
	LOXL3	↑ 50% ($p < 0.001$)

	AEBP1	↑ 50% ($p < 0.001$)
	VWA1	↑ 70% ($p < 0.001$)
	ITIH3	↑ 70% ($p < 0.001$)
Kern *et al.*, 2014 [29]	*mRNA expression*	
	Miscellaneous	
	IGF-1 Ea	↑ ($p < 0.005$)
	IGF-1 Eb	↑ ($p < 0.05$)
	IGF-1 Ec	↑ ($p < 0.005$)
	IGF-1 pan	↑ ($p < 0.005$)
Raue *et al.*, 2012 [36]	*mRNA expression*	
	Miscellaneous	
	ADAMTS12	Young: N/A<br>Older: ↑ 125%
	CLEC1A	Young: N/A<br>Older: ↑ 53%
	IGFBP2	Young: N/A<br>Older: ↑ 77%
	IGFBP7	Young: N/A<br>Older: ↑ 103%
	ITGB1BP3	Young: ↑ 161%<br>Older: N/A
	ITGA6	Young: N/A<br>Older: ↑ 73%

	LOX	Young: N/A<br>Older: ↑ 250%
	LOXL2	Young: N/A<br>Older: ↑ 103%
	MXRA5	Young: N/A<br>Older: ↑ 165%
	KDR	Young: N/A<br>Older: ↑ 63%
	TFPI2	Young: N/A<br>Older: ↑ 156%
Riedl *et al.*, 2010 [37]	*Number of tags per 100000 SAGE tags*	
	Miscellaneous	
	IGFN1	↑ 12
Robinson *et al.*, 2017 [38]	*mRNA expression*	
	Miscellaneous	
	ITGB2	HIIT young: (NS)<br>HIIT older: ↑67% ($p \leq 0.05$)<br>RET young: (NS)<br>RET older: (NS)<br>CT young: (NS)<br>CT older: (NS)

	INS-IGF2	HIIT young: (NS)
		HIIT older: ↑74% ($p \leq 0.05$)
		RET young: (NS)
		RET older: (NS)
		CT young: (NS)
		CT older: (NS)
	IGF2	HIIT young: (NS)
		HIIT older: ↑81% ($p \leq 0.05$)
		RET young: (NS)
		RET older: (NS)
		CT young: (NS)
		CT older: (NS)
Timmons *et al.*, 2010 [39]	*mRNA expression*	
	Miscellaneous	
	ADAMTS1	↑ 270%
	ADAMTS9	↑ 80%
	ADAMTSL3	↑ 50%
	COLEC12	↑ 80%
	IGF1	↑ 70-250%
	IGF2	↑ 220-320%
	IGFBP3	↑ 90-200%
	IGFBP4	↑ 50%
	IGFBP7	↑ 70-230%
	ITGA5	↑ 210%
	ITGA6	↑ 50-470%
	ITGB1	↑ 80%

	SMOC2	↑ 70%
	SPOCK1	↑ 60%
	TIMP1	↑ 230%
	TIMP2	↑ 50%
Valdivierso *et al.*, 2017 [40]	*Protein expression*	
	Miscellaneous	
	Vascular endothelial growth factor A	A/A alleles: (NS)<br>A/T alleles: ↑<br>T/T alleles: ↑
	mRNA expression	
	Miscellaneous	
	VEGF-R2	A/A alleles: ↑ 54%<br>A/T alleles: (NS)<br>T/T alleles: (NS)
	TIMP1	A/A alleles: (NS)<br>A/T alleles: (NS)<br>T/T alleles: ↑ 132%
	PGF	A/A alleles: (NS)<br>A/T alleles: (NS)<br>T/T alleles: ↑ 65%
	FGF R4	A/A alleles: (NS)<br>A/T alleles: (NS)<br>T/T alleles: ↑ 169%

Walton *et al.*, 2019 [41]	*mRNA expression*	
	Miscellaneous	
	MMP2	↑ 33.7% ($p = 0.032$)
	MMP14	↑ 60.5% ($p = 0.01$)
	TIMP2	↑ 26.4% ($p = 0.031$)
	HGF	↑ 43.7% ($p = 0.005$)
	IGF1	↑ 21.4% ($p = 0.035$)
	TGFβ1	↑ 39% ($p < 0.001$)
	LOX	↑ 36.3% ($p = 0.002$)
	SERPINE1	↑ 35% ($p = 0.04$)
